# Does Advanced Footwear Technology Improve Track and Road Racing Performance? An Explorative Analysis Based on the 100 Best Yearly Performances in the World Between 2010 and 2022

**DOI:** 10.1186/s40798-024-00683-y

**Published:** 2024-02-08

**Authors:** Steffen Willwacher, Patrick Mai, Janina Helwig, Markus Hipper, Burkay Utku, Johanna Robbin

**Affiliations:** Institute of Advanced Biomechanics and Motion Studies, Offenburg University of Applied Sciences, Campus West, Max-Planck-Str. 1, 77656 Offenburg, Germany

**Keywords:** Running, Sprinting, Running shoe, Running performance, Spikes, Sports performance, Locomotion

## Abstract

Advanced footwear technology (AFT) is currently being debated in sports. There is a direct evidence that distance running in AFT improves running economy. In addition, there is indirect evidence from competition performance for improved running performance from using AFTs in middle- and long-distance running and sprinting events. However, the extent to which world-class performance is affected across the full range of track and road racing events between genders has not been systematically analyzed. This study examined publicly available performance datasets of annual best track and road performances for evidence of potential systematic performance effects following the introduction of AFT. The analysis was based on the 100 best performances per year for men and women in outdoor events from 2010 to 2022, provided by the world governing body of athletics (World Athletics). We found evidence of progressing improvements in track and road running performances after the introduction of AFT for road races in 2016 and AFT for track racing in 2019. This evidence is more pronounced for distances longer than 1500 m in women and longer than 5000 m in men. Women seem to benefit more from AFT in distance running events than men. For the sprint events (100 m to 400 m hurdles), the peak performance gains in 2021 and 2022 compared to the pre-AFT period ranged from 0.6 to 1.1% and from 0.4 to 0.7% for women and men, respectively. For middle-distance events (400 m to 3000 m steeplechase), peak performance gains ranged from 0.6 to 1.9% and from 0.6 to 0.7% for women and men, respectively. For distances from 5000 m to the marathon, performance gains ranged from 2.2% to 3.5% and 0.7% to 1.4% for women and men, respectively. While the observational study design limits causal inference, this study provides a database on potential systematic performance effects after introducing advanced shoes/spikes in track and road running events in world-class athletes. Further research is needed to examine the underlying mechanisms and, in particular, potential gender differences in the performance effects of AFT.

**Supplementary Information:**

The online version contains supplementary material available at 10.1186/s40798-024-00683-y.

## Key points

- The study provides indirect evidence of the performance-enhancing effects of advanced footwear technology, particularly for longer distances and in women.
- Compared to before the introduction of advanced footwear technology, the greatest gains in performance were seen in long-distance events (up to 3.5% for women and 1.4% for men) and smaller gains in sprint and middle-distance events.
- However, limitations of the study should be considered, such as the observational nature of the analysis and potential confounding factors such as performance-enhancing drugs.

**Supplementary Information:**

The online version contains supplementary material available at 10.1186/s40798-024-00683-y.

## Introduction

Advanced footwear technologies (AFTs) have been the subject of intense debate in the sporting world in recent years. Critics see them as techno-doping that artificially enhances athletes' performance [1].

Thanks to advancements in materials science, athletic shoes have undergone rapid development, especially in recent years. Advanced, in the context of this article, means lightweight footwear technologies that use a compliant and resilient foam as the cushioning element in combination with curved stiffening elements running along the sole [2, 3]. In the case of spiked track running shoes, some manufacturers add a spike plate in the forefoot to which the spikes are attached [4].

Potential performance improvements of distance running shoes can be evaluated by assessing their effects on the running economy (RE). Improvements in RE measured in the laboratory translate directly to race performance, although the magnitude is smaller [5–7]. Overall, there is clear, direct evidence to suggest that AFT improves RE in distance running [7–9], even though these improvements might be running speed dependent [10] and differ between individuals [7, 11]. While some of these studies included world-class athletes [11], most were performed with high-caliber men.

The real-world success of AFTs for distance running across a wide range of performance levels was demonstrated in an analysis based on data from the social fitness network Strava. This analysis suggests that AFTs for road running provide a 3–4% advantage over "traditional" running shoes [12]. Evaluating performance between footwear conditions is more difficult for shorter running and sprinting distances. Here, energy is supplied much more by anaerobic metabolism, so an evaluation of RE-based solely on oxygen uptake is inappropriate [13, 14]. Nevertheless, it is still essential to maintain a high speed as efficiently as possible during long sprints and middle-distance races [15, 16].

In these situations, a viable option is to directly test performance while wearing different (spike) shoes via repeated test runs over the competition distance [4]. The performance criterion would then be the time achieved with each shoe. Methodologically, it is difficult to control for the underlying conditions that may influence the time achieved. These include influences such as motivation, fatigue (e.g., due to training on previous days, psychological stress), time of day, training effects, and sleep. Controlling for these conditions is challenging and virtually impossible, making it difficult to quantify the effects of footwear on track running performance in elite athletes [4]. Integrating world-class athletes in such a testing paradigm seems even more challenging, given their sophisticated training protocols, competition, public relations, and travel schedules.

An alternative approach would be to identify systematic changes in the best performances recorded in competitive events, assuming that AFTs were increasingly adopted after their introduction. Such an indirect approach has already been taken for road racing performances [17–20], providing evidence for performance improvements using AFTs. Recently, this type of analysis was also applied to sprint running performances, highlighting potential performance improvements using AFTs [21]. Notably, there was evidence of potentially greater improvement with AFT use in women than in men for both distance running and sprint performance [18, 19, 21]. While these studies provide valuable insights, no study has applied this approach to the full range of Olympic running events, from the 100 m to the marathon, while accounting for potential differences in AFT-induced performance improvements between events and genders.

Therefore, this study aimed to examine publicly available performance datasets of annual best track and road performances for evidence of potential systematic performance effects following the introduction of AFTs. In addition, the study aimed to determine whether there was evidence of differences in potentially AFT-induced performance improvements between genders and between distances.

We hypothesized that there is identifiable evidence for progressive improvements in track and road running performances after the introduction of AFTs for road running (in 2016) and AFTs for track running (in 2019). Based on previous findings [17–21], we hypothesized a performance improvement for AFTs for shorter and longer running distances and that women benefit more from introducing AFTs than men.

## Methods

We based our analysis on a publicly available database of the 100 best track and road running performances provided by the world governing body of athletics (World Athletics). We extracted the data from the World Athletics season top lists (www.worldathletics.org, accessed January 31, 2023). We considered the outdoor events of the top 100 men's and women's performances from 2010 to 2022. Because AFTs for road running were introduced earlier (2016) than AFTs for track running (2019), we used different baseline reference periods for comparison.

**Fig. 1:**
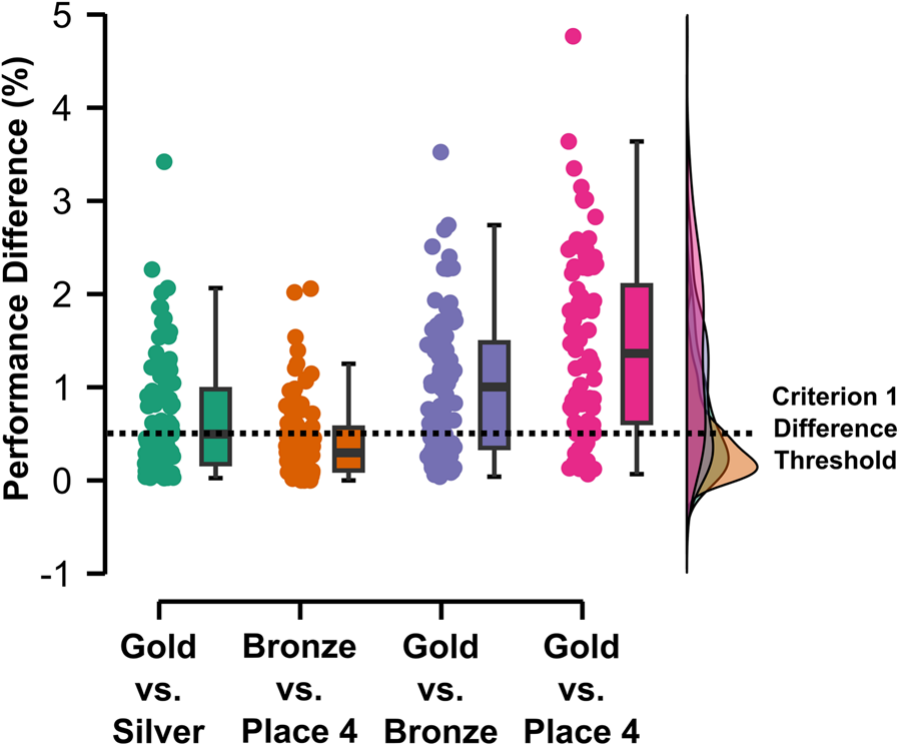
Differences between positions in track and marathon running events in the 2008 to 2021 Olympic Games

We defined the reference period from 2010 to 2015 for long distances (i.e., half-marathon and marathon) and from 2010 to 2018 for events from 100 to 10,000 m to represent the period before AFTs for distance running and track running were introduced, respectively. As a result, the observation periods for AFTs for road running and AFTs for track running began in 2016 and 2019, respectively. We excluded 2020 from the analysis because of the low number of races and different training timelines due to the Covid-19 pandemic. In order to assess the potential influence of AFTs on performance, we defined three criteria:

The first criterion was that the arithmetic mean of the medians of the 100 best performances per year during the observation period should be at least 0.5% faster than the reference value. The reference value was calculated as the mean of the medians of the reference period years (Fig. 1). The difference threshold (> 0.5%) was chosen to take into account the distributions of the differences obtained between the first places in the track and marathon events during the last four Olympic Games (Fig. 1).

The chosen threshold of 0.5% is above the median of the differences between gold and silver medals (0.497%) and well above the median of the differences between bronze medalists and fourth place (0.297%).

In addition to this criterion, we have added two criteria that rely more on arbitrary thresholds: the second criterion was met if at least 75% of the years in the observation period were statistically significantly faster than the reference value. We applied the Bonferroni correction of alpha levels to avoid alpha error accumulation [22]. The resulting alpha-level threshold was *p* < 0.000595. The year 2020 was again not considered due to the strong impact of the pandemic, as explained above. Due to the non-normal and non-symmetric distribution of the sample, we used the one-sample Wilcoxon signed-rank test to compare individual years with the reference value.

The final criterion was whether two years within the observation period were the fastest years within the entire analysis period overall.

In addition, to test for differences in performance improvement between events and genders, we performed a two-factor (gender and event) analysis of variance (ANOVA; alpha = 0.05) on the relative performance improvement compared to the respective reference value per event and gender for the years 2021 and 2022. Because the distribution of these relative performance improvements deviated significantly from a normal distribution, we performed the ANOVA analyses on the rank-transformed data [23].

## Results

**Fig. 2:**
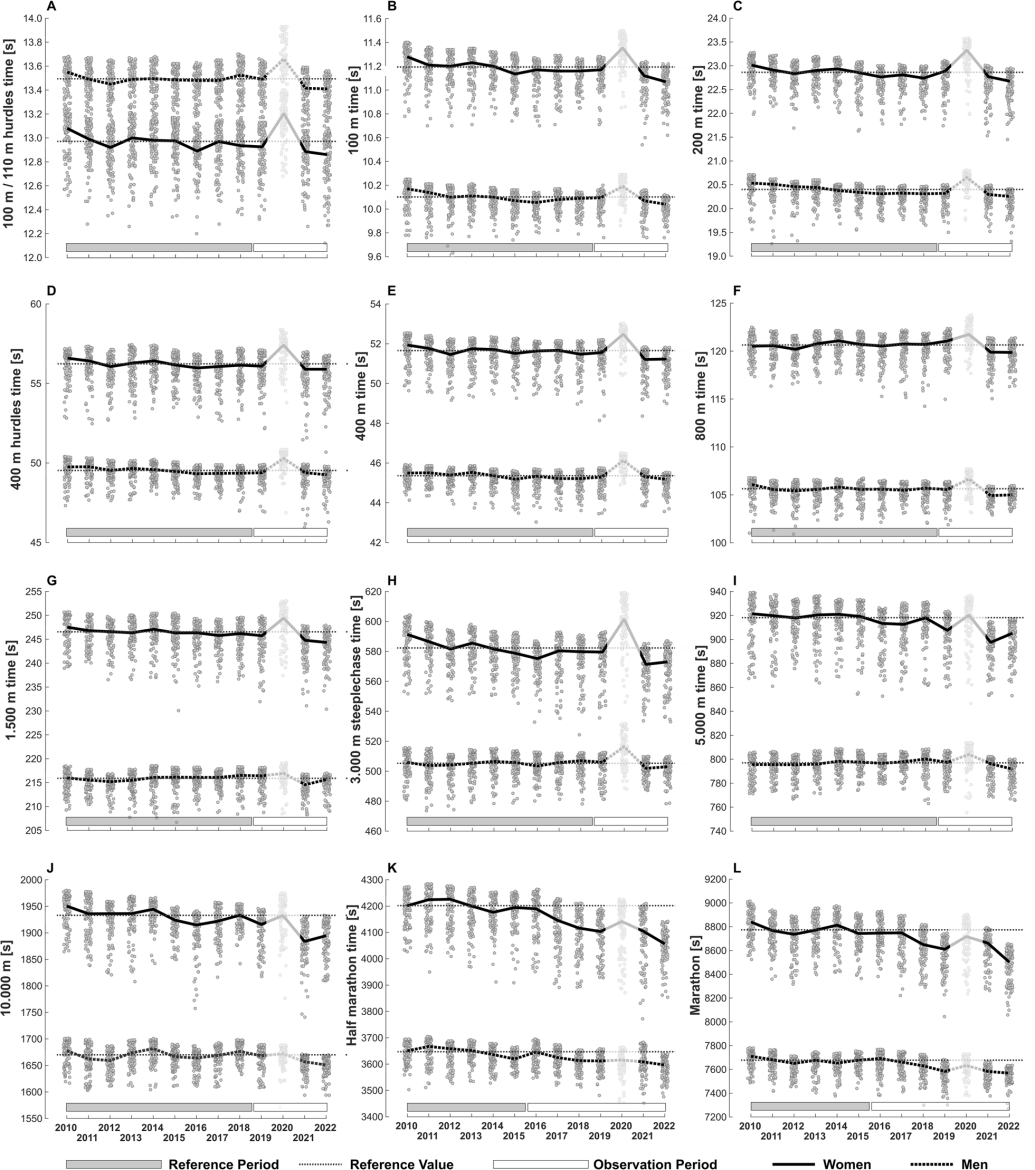
Evolution of the 100 best track and road performances between 2010 and 2022. Thick lines show the evolution of the median of the 100 best performances per year. The dotted horizontal line marks the reference value for each event (separately for men and women). The gray and white boxes highlight each event's reference and observation periods, respectively. The year 2020 was not included in the analysis

**Fig. 3:**
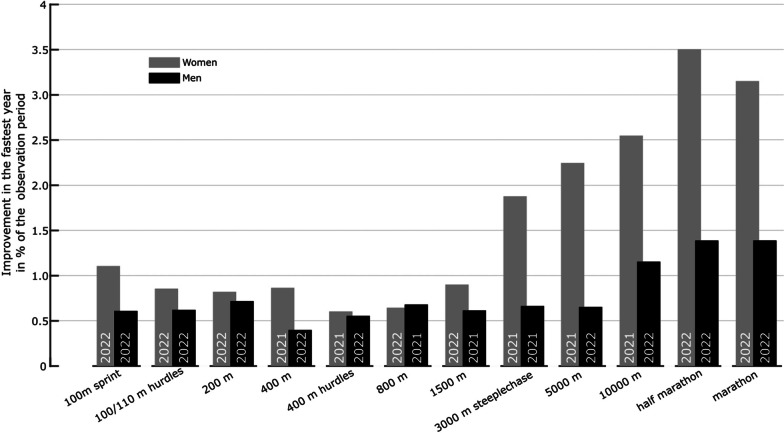
Difference between the performance of the best year in the observation period and the reference value (in %). The best year is highlighted in each bar

Based on our predefined criteria, we found evidence in several track and road events (Table 1, Fig. 2, and Fig. 3). Maximum performance increases within one year compared to the reference level ranged between 0.4 and 1.1% in track events up to 1500 m (Fig. 3). For longer running distances, larger maximum yearly performance increases to the reference of up to 3.5% (half-marathon, female athletes) were observed. However, the evidence for performance gains during the observation period compared to the pre-AFT period was more pronounced in women than men (Table 1, Figs. 2, and Fig. 3) in distances longer than 1500 m. Figure 2 shows the evolution of the top 100 track and road running performances yearly since 2010.

**Table 1:** Criteria table

	First criterion (%)		Second criterion (%*)		Third criterion (best two years)	
	Women	Men	Women	Men	Women	Men
100 m	**0.66**	0.33	**100**	**100**	**2022, 2021**	2022, 2016
100/110 m hurdles	**0.63**	0.41	**100**	67	**2022, 2021**	**2022, 2021**
200 m	0.37	**0.55**	67	**100**	2022, 2018	**2022, 2021**
400 m	**0.63**	0.22	**100**	**100**	**2021, 2022**	2022, 2015
400 m hurdles	0.49	0.38	67	**100**	**2021, 2022**	2022, 2016
800 m	0.31	0.46	67	67	**2022, 2021**	**2021, 2022**
1500 m	**0.66**	0.16	**100**	33	**2022, 2021**	2021, 2012
3000 m steeplechase	**1.33**	0.33	**100**	67	**2021, 2022**	**2021, 2022**
5000 m	**1.63**	0.24	**100**	33	**2021, 2022**	2022, 2012
10,000 m	**1.84**	**0.69**	**100**	67	**2021, 2022**	**2022, 2021**
Half-marathon	**2.05**	**0.84**	**100**	**86**	**2022, 2019**	**2022, 2021**
Marathon	**1.45**	**0.73**	**86**	71	**2022, 2019**	**2022, 2021**

A bold style indicates that a criterion has been met. First criterion: the mean of the medians of the 100 best performances per year during the observation period should be at least 0.5% faster than the reference value (% improvements are provided). Second criterion: at least 75% of the years in the observation period were statistically significantly faster than the reference value (% of years are provided). Third criterion: two years within the observation period were overall the fastest years within the entire analysis period

*Significance at *p* < 5.95 × 10^–4^

For the sprint events (100–400 m hurdles), the peak performance gains in 2021 and 2022 compared to the pre-AFT period ranged from 0.6% to 1.1% and 0.4% to 0.7% for women and men, respectively (Fig. 3). For middle-distance events (400–3000 m steeplechase), peak performance gains ranged from 0.6% to 1.9% and 0.6% to 0.7% for women and men, respectively (Fig. 3). For distances from 5000 m to the marathon, performance gains ranged from 2.2% to 3.5% and 0.7% to 1.4% for women and men, respectively. (Fig. 3).

For women, all three criteria were met in all events except the 200 m, 400 m hurdles, and 800 m (Table 1). For men, all three criteria were met only for the half-marathon. However, all but the third criterion was met for the 10,000 m and marathon distances (Table 1).

**Fig. 4:**
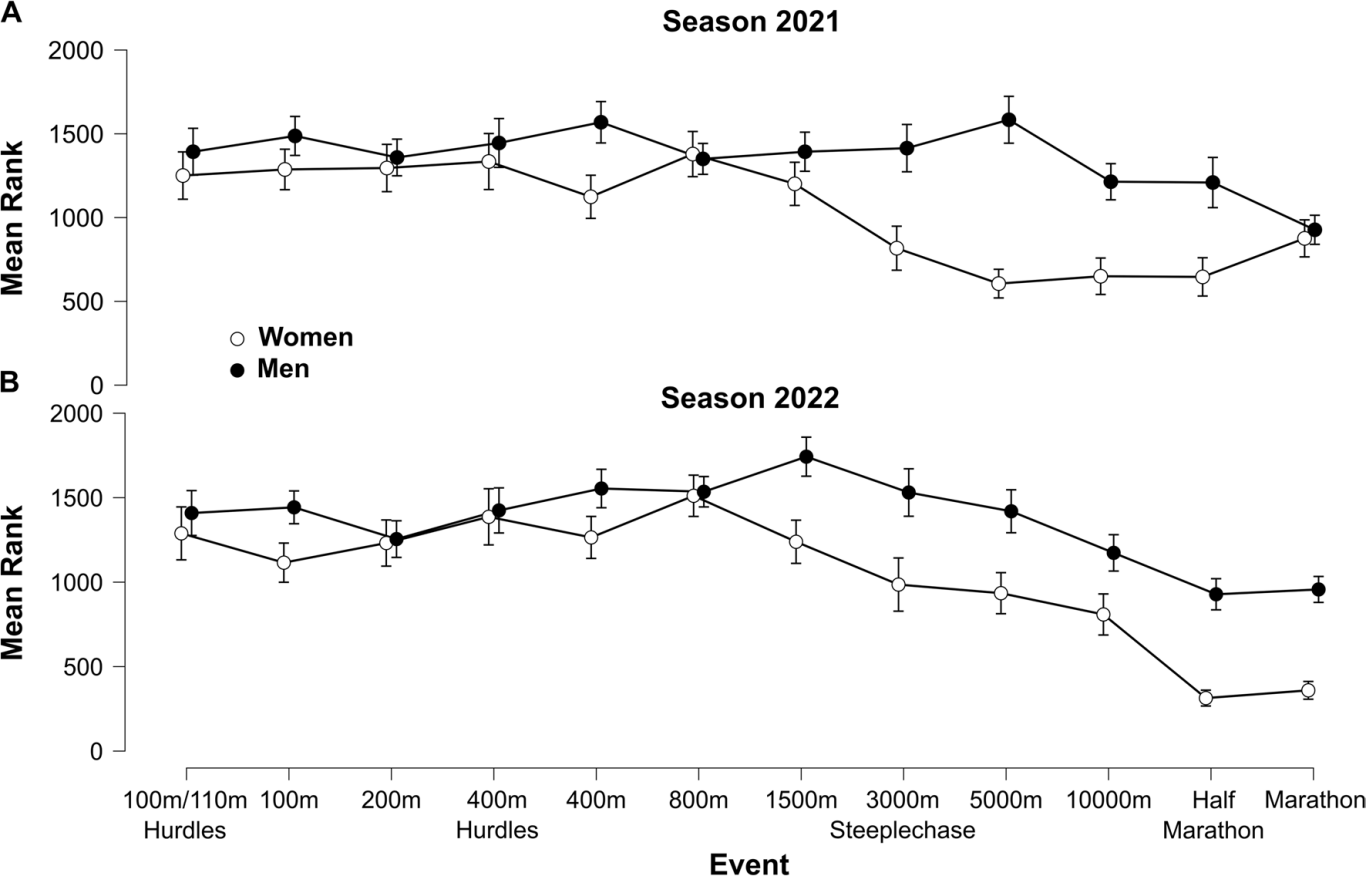
Results of the rank-transformed two-factor (events, gender) analysis of variance (ANOVA) for the years 2021 (**A**) and 2022 (**B**). Lower ranks indicate a more pronounced performance improvement compared to the respective reference value. Vertical error bars indicate 95% confidence intervals for the respective means

Furthermore, the ANOVA analyses revealed highly significant (*p* < 0.001) events by gender interaction effects, both for performance improvements in the years 2021 and 2022 compared to the respective reference values (Fig. 4). The interaction effects likely resulted from the more pronounced differences in the longer running distances (> 800 m), as well as 100 m and 400 m (Fig. 4).

## Discussion

The present study aimed to explore indirect evidence of potential performance improvements induced by the introduction of AFTs for road and track running from the 100 yearly best race times in the world. The findings suggest evidence of a performance effect of AFTs in both men and women, but the effect appears to be more pronounced in women. The largest improvements were observed in distances longer than 5000 m for men and longer than 1500 m for women. These findings suggest that our general hypothesis that AFTs positively affect running performance across athletic track and road racing events can be accepted. However, this general finding requires a more differentiated analysis.

Our results suggest that the performance effect of AFTs may be more pronounced in women than in men. The causes of this phenomenon are uncertain, but it may be due to factors such as differences in body mass, competition running speeds, or running biomechanics between men and women. Lower body weight, lower running speed, and increased stride frequency in women [24] result in reduced absolute ground reaction force generation, while longer ground contact times, also reported in women [24], may increase ground reaction forces. It is currently not well reported whether shoe companies scale the stiffness of the elastic cushioning foams or the bending stiffness of the carbon elements to the mass, speed, or gender of runners. However, assuming that this scaling does not occur in most AFTs, it can be assumed that women's specific anthropometrics and biomechanics appear to benefit more from current AFT designs. The observed performance improvements in women's running might also be partially attributed to increasing professionalization, as reflected in more racing opportunities and reduced prize money disparities. Additionally, decreasing cultural and religious barriers to women's sports participation might have expanded the talent pool, intensifying competition. These sociocultural factors, alongside technological advancements, might contribute to the advancements in the running and sprinting performances of women.

Another design feature partially linked to the performance-enhancing effects of AFTs is the longitudinal stiffening element embedded in the midsole. The bending stiffness of athletic footwear can affect the biomechanics of running and, therefore, RE and sprint performance [3, 25–28]. Optimal bending stiffness depends on body mass [27] and running speed [29, 30] and may differ between athletes with different strength abilities [25]. In addition to the bending stiffness, the geometry of the stiffening element appears to be critical in moderating running biomechanics and performance. While the upwardly curved shape of the stiffening element has motivated the concept of a rocker mechanism [31], it has also been shown that the performance-enhancing effects of an AFT remain even after the bending stiffness of the midsole is removed, highlighting that the compression characteristics of AFTs likely affect RE more than the bending stiffening element [32]. How the bending stiffness component interacts with the compression behavior of the highly elastic foam materials in the midsole to improve performance at the individual level is not well understood at this time. Consequently, large interindividual differences in performance benefits with using AFTs have been reported [11]. A better understanding is needed to design optimal footwear technologies for individual groups of runners, such as men and women, shorter and taller runners, or faster and slower runners. Consequently, future studies will need to look more closely at why women might benefit more from AFTS or if other factors can explain why world-class performances in distance running events have improved more for women than men since the introduction of super running shoes.

Upon analyzing the effects of AFTs on performance changes, it is evident that the potential improvements are more pronounced for longer track distances compared to sprinting distances (as shown in Figs. 2 and 3). Several reasons might explain this observation. Firstly, incorporating softer and more elastic materials into the forefoot of sprint shoes may not lead to performance-enhancing effects to the same extent as in distance running shoes. This difference in response to softer and more elastic materials in the forefoot might be because sprinting requires generating large and well-directed forces to the ground during short ground contact times [33, 34]. This force application might be affected by introducing foam materials to the forefoot. There is a lack of systematic investigation into how different forefoot foam properties and geometries interact with (carbon fiber) stiffening elements and how this interaction contributes to performance in sprint events. Secondly, AFTs for sprinting have not been available for as long as those for distance running. Consequently, they may have been less widely distributed among the world-class sprinting community. Athletes may not have been as familiar with this new technology as distance runners who have been using AFTs longer. It is not known whether runners need to adjust their running mechanics (e.g., foot strike pattern, stride length, or vertical center of mass oscillation) to maximize the performance-enhancing effects of AFTs. Longitudinal studies conducted during the transition from traditional athletic footwear to AFTs may shed light on this question in the future. Finally, the recent introduction of AFTs for sprinting and the lack of biomechanical studies on their mechanisms of potential performance enhancement suggest that these shoes may still evolve in their functional design, and larger performance improvements enabled by this technology may be possible in the future.

While our study provides indirect evidence for the performance effect of AFTs, several limitations need to be considered. The study is observational and does not account for other factors that may have influenced performance improvements, such as changes in the use of performance-enhancing drugs (PEDs), training methods, pacing strategies [35], environmental conditions, the presence of Olympic Games in the same year, or recovery effects due to the Covid-19 pandemic. In addition, we did not check whether AFTs were actually worn in the 100 best performances; therefore, it is uncertain whether AFTs actually affected performance in all races during the observation period. This uncertainty may have led to an underestimation of the observed performance effects. In addition, the observation period is relatively short, particularly for AFTs for track running, which limits the amount of data available to analyze the impact of AFTs in track events. Therefore, it is recommended that this analysis be updated when more years of data are available. Furthermore, the criteria used to identify potential performance improvements are arbitrary, even though we based them on the distribution of actually relevant performance differences (criterion 1), statistical differences (criterion 2), and overall performance improvement (criterion 3). In addition, the results of the criterion-based analysis are consistent with the results of the ANOVA analyses. Another limitation is that the observation periods for track and road events were different due to the later introduction of AFTs for track events. This difference may have affected comparisons between events. Future studies could include more balanced and longer observation periods to estimate the effects of AFTs on performance more robustly. Next to applying indirect race performance analyses to understand the impact of AFTs, future studies should provide more direct evidence by performing experiments that allow for deriving direct cause-effect relationships.

Regarding PED use, there is evidence that reduced testing during the COVID-19 pandemic may have made it easier for athletes to use PEDs without being detected. This lack of testing could have contributed to performance improvements in 2021 and 2022 [36–38]. In addition, world-class athletes may have generally relied more heavily on using PEDs in the observation compared to the baseline period. This behavior would undermine our assumption that the performance gains in the observation period were primarily due to AFTs. New developments in PED testing and their potential retrospective application to the periods considered in this study may better address this issue in the future.

In theory, performance improvements could have been achieved through improved training methods, which could have resulted from, e.g., a broader application of scientific knowledge in training practice. Improvements might relate to improving RE, training intensity, recovery, or load management in general. Whether these factors have influenced our analysis cannot be determined from the information available. One aspect often mentioned anecdotally by runners is that the soft cushioning of AFTs for distance running may affect muscle damage and, therefore, recovery times. There is preliminary evidence that AFTs may reduce muscle damage and neuromuscular fatigue after intense races or training sessions [39, 40]. On the other hand, Black et al. showed that the use of an AFT did not significantly reduce markers of muscle damage after a prolonged downhill run compared to traditional running shoes, although RE was improved with the AFT in both the absence and presence of muscle damage [41]. Despite these recent results, the hypothesis that softer cushioning would affect recovery times and, therefore, allow for higher training volumes or intensities needs to be tested in more detail in the future.

## Conclusion

In conclusion, our study provides indirect evidence for the performance-enhancing effects of AFTS for distance and track running, which appear to be more pronounced in women and at longer running distances. However, several limitations need to be considered, including the study's observational nature, the potential influence of PEDs, and the uncertain impact of other factors such as training methods and recovery effects. Future studies should address these limitations and provide a more direct basis for the potential performance enhancements due to AFTs. Nevertheless, our findings have important implications for the design and use of AFTs in competitive distance running, particularly concerning gender differences in footwear design. It seems crucial for manufacturers, coaches, and athletes to consider the specific anthropometrics and biomechanics of runners when designing and selecting AFTs and to continue to monitor the effects of new footwear technologies on athletic training and performance.

### Supplementary Information

**Additional file 1**. Supplementary tables.

## Data Availability

All extracted information can be found in the figures and tables. The raw data of the study are provided as an online digital supplement (Additional file 1).

## Abbreviations

RE: Running economy
PEDs: Performance-enhancing drugs
AFT: Advanced footwear technology
AFTs: Advanced footwear technologies
